# Natural selection at the RASGEF1C (GGC) repeat in human and divergent genotypes in late-onset neurocognitive disorder

**DOI:** 10.1038/s41598-021-98725-y

**Published:** 2021-09-28

**Authors:** Z. Jafarian, S. Khamse, H. Afshar, H.R. Khorram Khorshid, A. Delbari, M. Ohadi

**Affiliations:** 1 Iranian Research Center on Aging, University of Social Welfare and Rehabilitation Sciences, Tehran, Iran; 2 Personalized Medicine and Genometabolomics Research Center, Hope Generation Foundation, Tehran, Iran

**Keywords:** Evolution, Genetics, Molecular biology, Neuroscience

## Abstract

Expression dysregulation of the neuron-specific gene, *RASGEF1C* (RasGEF Domain Family Member 1C), occurs in late-onset neurocognitive disorders (NCDs), such as Alzheimer’s disease. This gene contains a (GGC)13, spanning its core promoter and 5′ untranslated region (RASGEF1C-201 ENST00000361132.9). Here we sequenced the (GGC)-repeat in a sample of human subjects (N = 269), consisting of late-onset NCDs (N = 115) and controls (N = 154). We also studied the status of this STR across various primate and non-primate species based on Ensembl 103. The 6-repeat allele was the predominant allele in the controls (frequency = 0.85) and NCD patients (frequency = 0.78). The NCD genotype compartment consisted of an excess of genotypes that lacked the 6-repeat (divergent genotypes) (Mid-P exact = 0.004). A number of those genotypes were not detected in the control group (Mid-P exact = 0.007). The *RASGEF1C* (GGC)-repeat expanded beyond 2-repeats specifically in primates, and was at maximum length in human. We conclude that there is natural selection for the 6-repeat allele of the *RASGEF1C* (GGC)-repeat in human, and significant divergence from that allele in late-onset NCDs. STR alleles that are predominantly abundant and genotypes that deviate from those alleles are underappreciated features, which may have deep evolutionary and pathological consequences.

## Introduction

Short tandem repeats (STRs), also known as microsatellites/simple sequence repeats, are an important source of evolutionary and pathological processes^1–5^. Numerous STRs within gene regulatory regions may have a link with the evolution of human and non-human primates through various mechanisms, such as gene expression regulation^6–9^. In a number of instances, there are indications of a link between STRs and late-onset neurocognitive disorders (NCDs) in human such as Alzheimer’s disease (AD) and Parkinson’s disease (PD)^10–12^.

*RASGEF1C* (RasGEF Domain Family Member 1C), located on chromosome 5q35.3, contains a (GGC)-repeat of 13-repeats, spanning its core promoter and 5′ UTR (RASGEF1C-201 ENST00000361132.9)^13^. Based on Ensembl 103 (ensemble.org), the transcript containing the (GGC)-repeat is at the highest support level annotated for the transcript isoforms of this gene (TSL:1). The protein encoded by *RASGEF1C* is a guanine nucleotide exchange factor (GEF) (https://www.genecards.org/cgi-bin/carddisp.pl?gene=RASGEF1C), and primarily interacts with a number of the ZNF family members, such as ZNF507, ZNF235, ZNF25, and ZNF612 (https://version11.string-db.org/cgi/network.pl?taskId=QkiV955revRw). In human, *RASGEF1C* is predominantly expressed in the brain (https://www.proteinatlas.org/ENSG00000146090-RASGEF1C/tissue), and aberrant regulation of this gene occurs in late-onset NCDs, such as AD^14^.

Abundancy of polymorphic CGG repeats in the human genome suggest a broad involvement in neurological diseases^15^. Large GGC expansions are strictly linked to neurological disorders of predominant neurocognitive impairment, such as fragile X-associated tremor and ataxia syndrome, NIID, and oculopharyngeal muscular dystrophy^16–21^.

Here we sequenced the *RASGEF1C* (GGC)-repeat in a sample of humans, consisting of late-onset NCDs and controls. We also analyzed the status of this STR across several primate and non-primate species.

## Materials and methods

### Subjects

Two hundred sixty-nine unrelated Iranian subjects of ≥ 60 years of age, consisting of late-onset NCD patients (n = 115) and controls (n = 154) were recruited from the provinces of Tehran, Qazvin, and Rasht. In each NCD case, the Persian version^22^ of the Abbreviated Mental Test Score (AMTS)^23^ was implemented (AMTS < 7 was an inclusion criterion for NCD), medical records were reviewed in all participants, and CT-scans were obtained where possible. Furthermore, in a number of subjects, the Mini-Mental State Exam (MMSE) Test^24^ was implemented in addition to the AMTS. A score of < 24 was an inclusion criterion for NCD.

The AMTS is currently one of the most accurate primary screening instruments to increase the probability of NCD^25^. The Persian version of the AMTS is a valid cognitive assessment tool for older Iranian adults, and can be used for NCD screening in Iran^22^.

The control group was selected based on cognitive AMTS of > 7 and MMSE > 24, lack of major medical history, and normal CT-scan where possible. The cases and controls were matched based on age, gender, ethnicity, and residential district. The subjects' informed consent was obtained (from their guardians where necessary) and their identities remained confidential throughout the study. The research was approved by the Ethics Committee of the Social Welfare and Rehabilitation Sciences, Tehran, Iran, and was consistent with the principles outlined in an internationally recognized standard for the ethical conduct of human research. All methods were performed in accordance with the relevant guidelines and regulations.

### Allele and genotype analysis of the *RASGEF1C* (GGC)-repeat

Genomic DNA was obtained from peripheral blood using a standard salting out method. PCR reactions for the amplification of the *RASGEF1C* (GGC)-repeat were set up with the following primers.

**Forward:** GAGGGTGAACTGGGTTTTGG.

**Reverse:** ACTCTAGCGGCTGAAAGAAG.

PCR reactions were carried out with a GC-TEMPase 2 × master mix (Amplicon) in a thermocycler (Peqlab-PEQStar) under the following conditions: touchdown PCR: 95 °C for 5 min, 20 cycles of denaturation at 95 °C for 45 s, annealing for 45 s at 67 °C (− 0.5 decrease for each cycle) and extension at 72 °C for 1 min, and 30 cycles of denaturation at 95 °C for 40 s, annealing at 57 °C for 45 s and extension at 72 °C for 1 min, and a final extension at 72 °C for 10 min. Genotyping of every sample included in this study was performed following Sanger sequencing by the forward primer, using an ABI 3130 DNA sequencer (Supplementary Information 1 and 2).

### Analysis of the *RASGEF1C* (GGC)-repeat across vertebrates

Ensembl 103 (https://www.ensembl.org/index.html) was used to analyze the interval between + 1 and + 100 of the TSS of the *RASGEF1C* in all the species in which this gene was annotated and the relevant region was sequenced. The CodonCode Aligner (https://www.codoncode.com) and Ensembl alignment programs (http://www.ensembl.org) were implemented for the sequence alignments across the species.

### Statistical analysis

The P-values were calculated using the Two-by-Two Table of the OpenEpi calculator (https://www.openepi.com/TwobyTwo/TwobyTwo.htm)^26^.

### Statement of ethics

The subjects' informed consent was obtained (from their guardians where necessary) and their identities remained confidential throughout the study. The research was approved by the Ethics Committee of the University of Social Welfare and Rehabilitation Sciences, Tehran, Iran, and was consistent with the principles outlined in an internationally recognized standard for the ethical conduct of human research.

## Results

### Predominant abundance of the *RASGEF1C* (GGC)6 in human.

**Figure 1:**
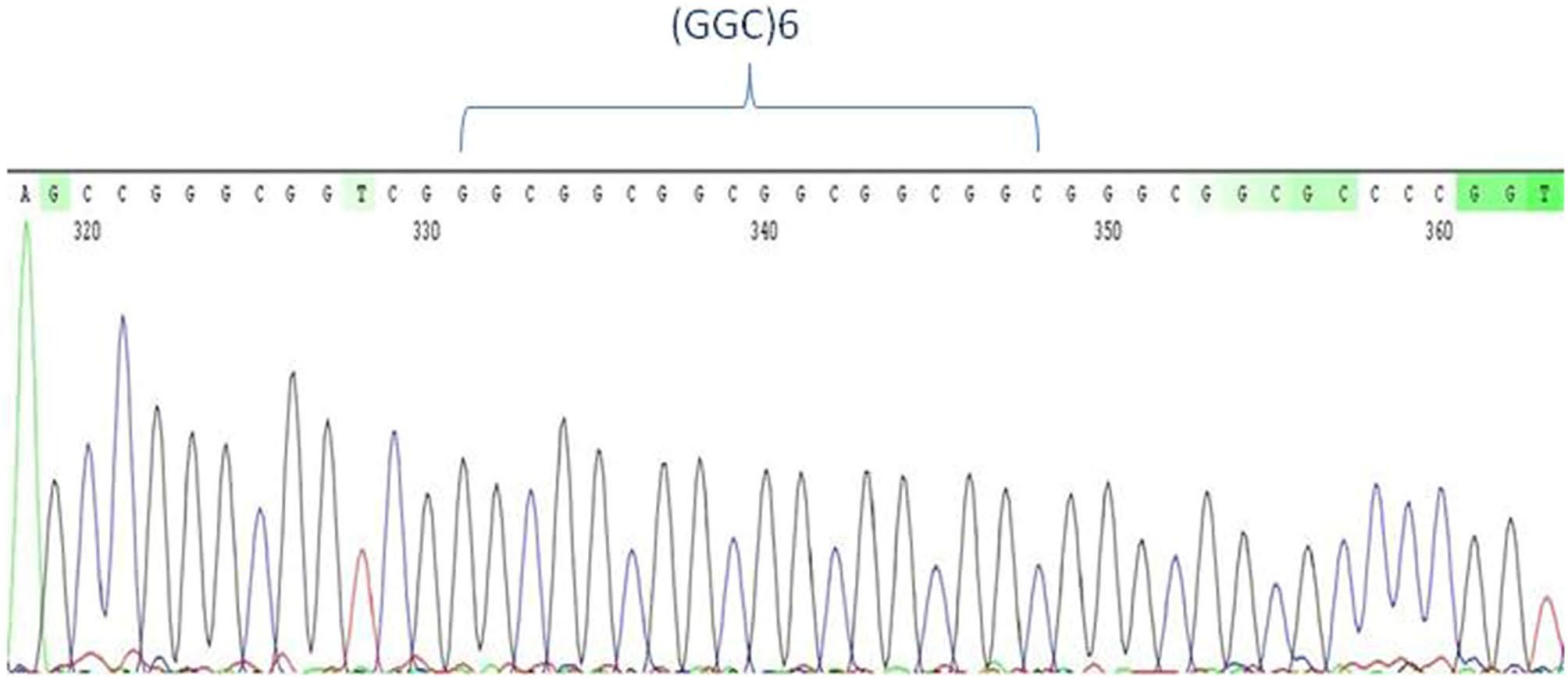
Electropherogram of the predominantly abundant allele at 6-repeats in the human *RASGEF1C* gene, in the context of a 6/6 genotype.

**Figure 2:**
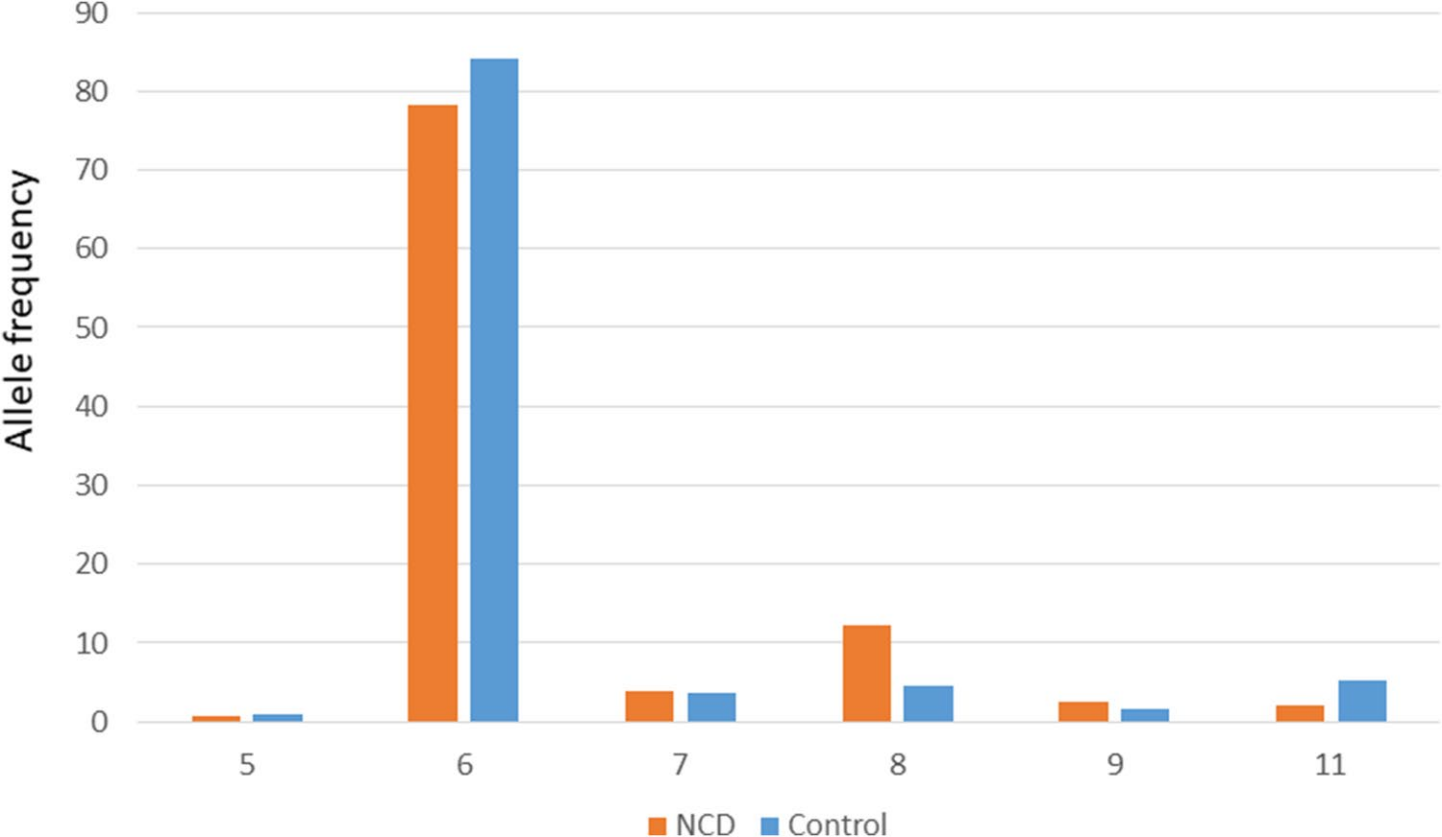
Allele frequency of the *RASGEF1C* (GGC)-repeat in NCD patients and controls. The 6-repeat was the predominant allele in both groups.

We detected six alleles at 5, 6, 7, 8, 9, and 11-repeats, of which the predominant allele was the 6-repeat (Figs. 1 and 2). The frequency of (GGC)6 was at 0.85 and 0.78 in the controls and NCD group, respectively (Fig. 2). At significantly lower frequencies, the 8 and 11 repeats ranked next in the NCD group and controls, respectively.

### Significant enrichment of divergent genotypes (genotypes that lacked the 6-repeat) in the NCD group

**Figure 3:**
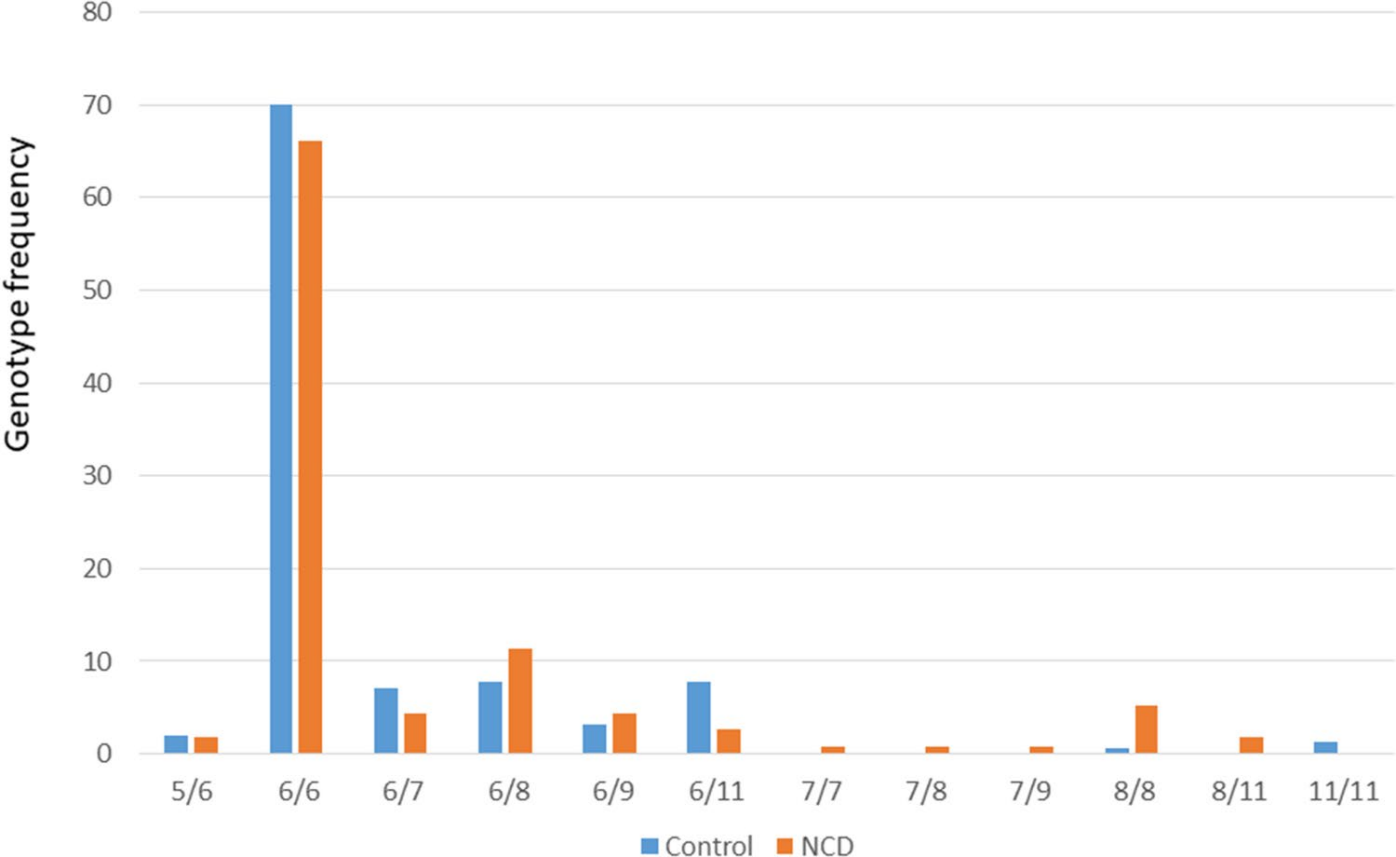
Genotype frequency of the *RASGEF1C* (GGC)-repeat in NCD patients and controls. While the 6/6 genotype was the predominant genotype in both groups, excess of divergent genotypes was detected in the NCD group.

**Figure 4:**
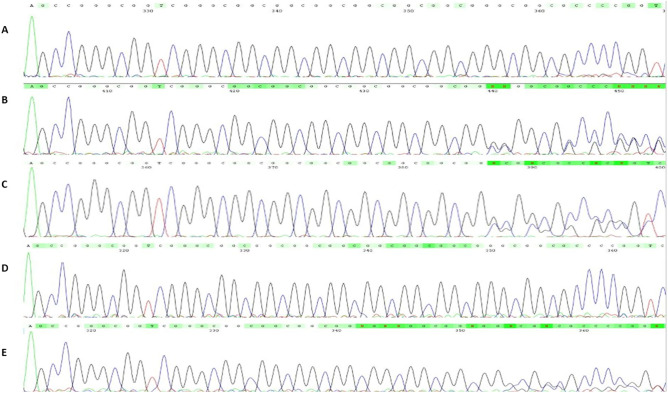
Electropherogram of the non-6 genotypes (divergent genotypes) at the *RASGEF1C* (GGC) in NCD patients. (**A**) 7/7, (**B**) 7/8, (**C**) 7/9, (**D**) 8/8, (**E**) 8/11.

We detected significant enrichment of genotypes that lacked the 6-repeat allele in the NCD group. Eleven out of 115 patients harbored such genotypes (Mid-P exact = 0.004) (Table 1, Figs. 3 and 4), whereas 3 out of 154 controls harbored those (p = 0.05). The divergent genotypes consisted of the 7, 8, 9 and 11 repeat alleles, and heterozygous and homozygous genotypes were detected in that genotype compartment.

**Table 1:** NCD patients and controls harboring divergent genotypes (lacking the 6-repeat).

Number	Sex	Age (years)	STR formula	AMTS	MMSE
**NCDs**					
1	F	87	7/7^a^	2	–
2	F	62	7/8^a^	5	19
3	M	72	7/9^a^	2	16
4	F	70	8/8	4	–
5	F	77	8/8	4	–
6	F	67	8/8	4	–
7	F	73	8/8	5	–
8	F	63	8/8	5	–
9	M	79	8/8	1	–
10	F	79	8/11^a^	3	18
11	M	60	8/11^a^	5	21
**Controls**					
1	F	78	11/11	9	–
2	M	70	11/11	10	–
3	M	73	8/8	10	–

*F* Female, *M* male.

^a^Disease-only genotypes.

Among the divergent genotypes, in 5 patients (4% of the NCD group) we detected genotypes that were not detected in the control group (hence the term “disease-only”) (Mid-P exact = 0.007) (Table 1, Fig. 4).

Patients harboring the divergent genotypes spanned a wide age range, between 60 to 78 years, and revealed moderate to severe neurocognitive dysfunction. Possible diagnoses also varied, such as AD in patients 1, 5, 9, and 10 and vascular dementia in patients 2 and 11.

In line with a higher frequency of the 8-repeat in the NCD group, we found a significant excess of the 8/8 genotype in this group in comparison to the control group (Mid-P exact = 0.01).

Although not statistically significant (p = 0.05), two control individuals harbored the 11/11 genotype, which was not detected in the NCD group (Table 1). The frequency of the 11-repeat allele was also found to be higher in the controls vs. NCDs.

### *RASGEF1C* (GGC)-repeat expanded specifically in primates, and was at maximum length in human

**Figure 5:**
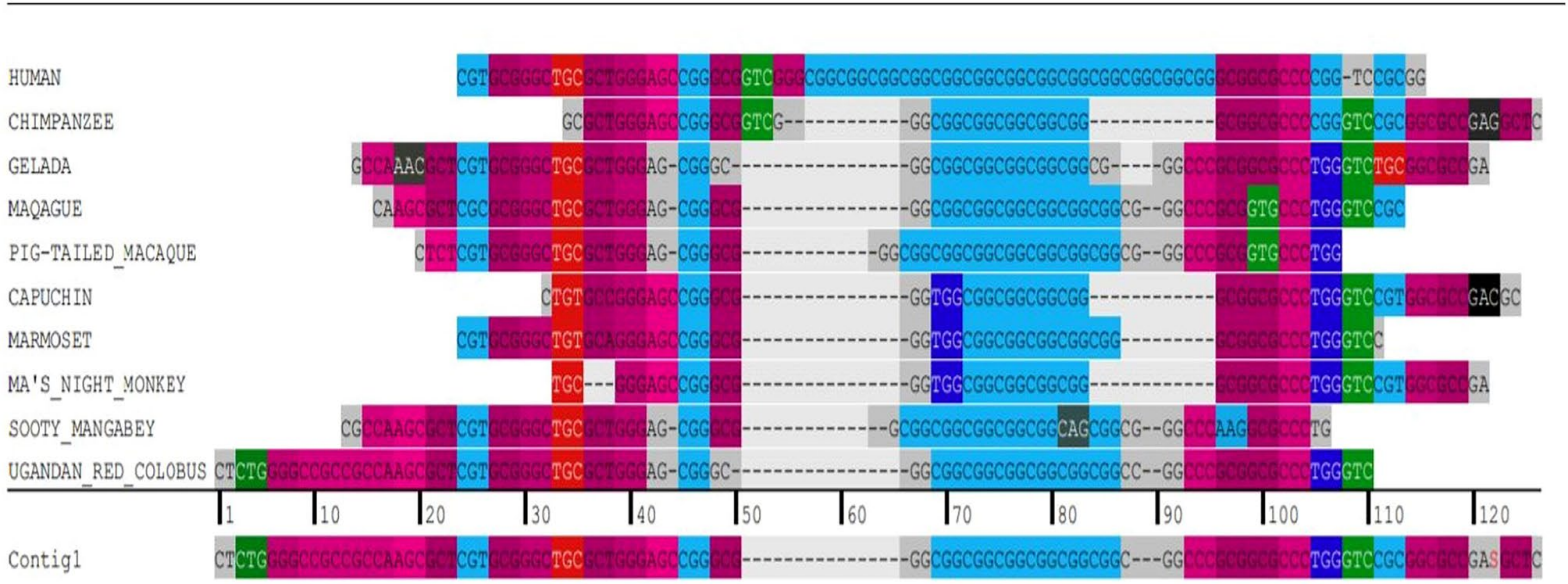
Sequence alignment of the *RASGEF1C* (GGC)-repeat across primate species, using CodonCode. The (GGC)-repeat was at maximum length in human.

**Figure 6:**
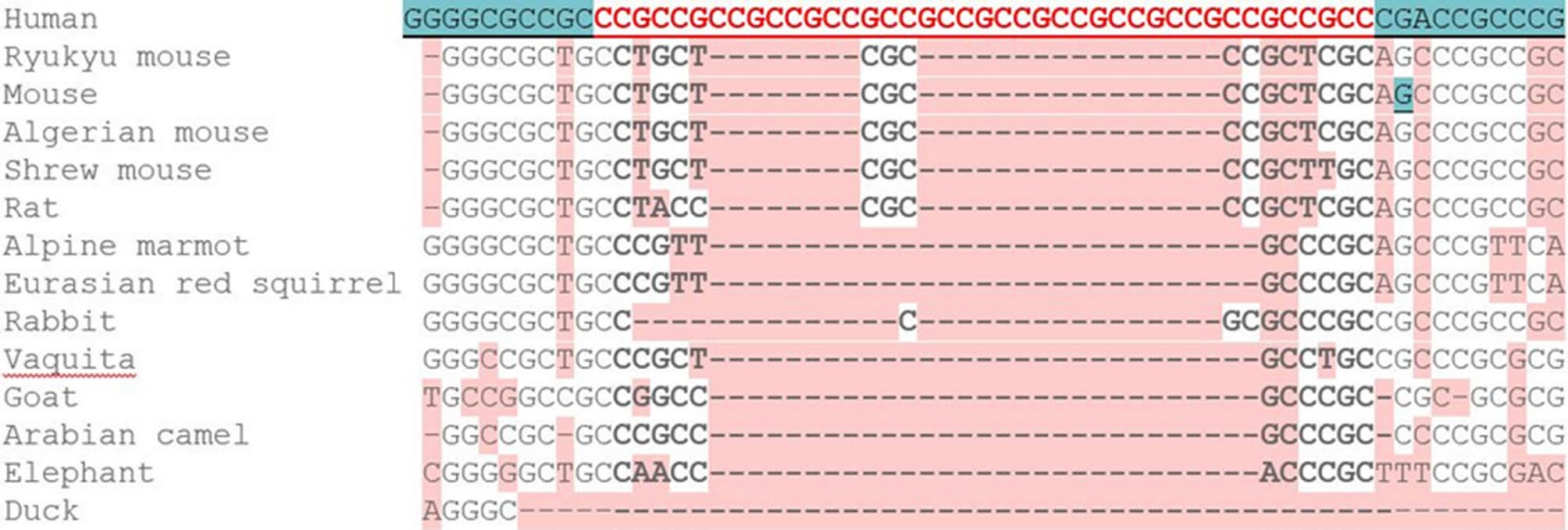
Sequence alignment of the *RASGEF1C* (GGC)-repeat across non-primates, using the Ensembl alignment program. Human is depicted as the reference sequence. Reverse strand depicted. (GGC)-repeat of > 2-repeats was not detected in non-primates.

Across all the species studied, the (GGC)-repeat was at maximum length in human. While in primates the minimum repeat length was 4-repeats (Fig. 5), the maximum length of (GGC)-repeat detectable in non-primates was 2-repeats (Fig. 6), indicating that this STR expanded specifically in primates.

## Discussion

We propose that there is natural selection for the 6-repeat of the *RASGEF1C* (GGC)n in human. This proposition is not only based on the predominant abundance of the 6-repeat allele in the human subjects studied, but also the significant enrichment of divergent genotypes, lacking this allele in the NCD compartment. A number of divergent genotypes were detected in the NCD group only. Evidence of natural selection for an abundant allele in human has been previously reported by our group in the instance of the exceptionally long CA-repeat in the core promoter of the human *NHLH2* gene, and enrichment of genotypes lacking the predominantly abundant allele (the 21-repeat) in patients afflicted with late-onset NCD^1^. It is commonly assumed that genes influencing health in later life are not subject to natural selection. However, findings on the *APOE* alleles and several other NCD susceptibility loci^27,28^ indicate that natural selection indeed happens on such alleles. *RASGEF1C* may also be linked to other yet to be identified phenotypes, which may impose natural selection at the (GGC)-repeat.

Based on the AceView database^29^, in comparison with several primates, the brain expression of *RASGEF1C* has the least quantile expression level in human (https://www.ncbi.nlm.nih.gov/IEB/Research/Acembly), which coincides with maximum length of the (GGC)-repeat in human vs. all other primates. A (GGC)-repeat of similar length range in another gene, *Reelin* (*RELN*), which is critical to neurodevelopment in human, shows declining luciferase activity with increasing GGC repeat number^30^. However, it should be noted that the *RASGEF1C* (GGC)-repeat is among a number of other regulatory factors, which may also affect gene expression, and a link can only be established through future studies.

While aberrant repeat associated non-AUG translation (RAN), DNA hypermethylation, and polyglycine protein translation are established mechanisms linked to large (GGC)-repeat expansions (> 100 repeats) in a spectrum of neurological disorders in human^5,31,32^, the mechanisms underlying repeat selection at the range of the *RASGEF1C* (GGC)-repeat and also its link to late-onset NCD need to be clarified in the future studies.

Despite the high prevalence and debilitating characteristics of late-onset NCDs, genetic studies in this group of disorders have resulted in a number of genes with mild to modest effect for the most part^33^. In a novel approach, we selected the patients group based on late-onset NCD as an entity, without differentiating the NCD subtypes. The advantage of this approach was to eliminate the often-ambiguous diagnoses made for the NCD subtypes, which frequently co-occur and overlap in respect of the clinical and pathophysiological manifestations^34–36^, and are associated with “probable” and “possible” conclusions for the most part (DSM-5).

The *RASGEF1C* (GGC)-repeat expanded beyond 2-repeats in primates, and was at maximum length in human. It may be speculated that this locus participates in characteristics and phenotypes that have dramatically diverged in human, such as the higher order brain functions.

Our data warrant further functional studies on the (GGC)-repeat and sequencing this repeat in larger sample sizes and various human populations afflicted with major neurological disorders.

## Conclusion

We provide a pilot study on repeat length selection at the human *RASGEF1C* (GGC)-repeat, at 6-repeats, and significant enrichment of genotypes lacking this allele in patients with late-onset NCD. Indication of natural selection for predominantly abundant STR alleles and divergent genotypes unfold a previously underappreciated feature of STRs in human evolution and disease.

## Supplementary Information

Supplementary Information 1.

Supplementary Information 2.

Supplementary Legends.

## Data Availability

Raw data are available in Supplementary Information 1 and 2.

## Abbreviations

AD: Alzheimer’s disease
AMTS: Abbreviated Mental Test Score
MMSE: Mini-Mental State Exam
NCD: Neurocognitive disorder
RASGEF1C: RasGEF Domain Family Member 1C
STR: Short tandem repeat
TSS: Transcription start site
UTR: Untranslated region

## References

[1] Afshar H, Adelirad F, Kowsari A, Kalhor N, Delbari A, Najafipour R, Foroughan M, Bozorgmehr A, Khamse S, Nazaripanah N, Ohadi M (2020). Natural selection at the NHLH2 core promoter exceptionally long CA-repeat in human and disease-only genotypes in late-onset neurocognitive disorder. Gerontology.

[2] Sulovari A, Li R, Audano PA, Porubsky D, Vollger MR, Logsdon GA, Warren WC, Pollen AA, Chaisson MJP, Eichler EE, Human Genome Structural Variation Consortium (2019). Human-specific tandem repeat expansion and differential gene expression during primate evolution. Proc. Natl. Acad. Sci. U. S. A..

[3] Flynn JM, Caldas I, Cristescu ME, Clark AG (2017). Selection constrains high rates of tandem repetitive DNA mutation in Daphnia pulex. Genetics.

[4] Watts PC, Kallio ER, Koskela E, Lonn E, Mappes T, Mokkonen M (2017). Stabilizing selection on microsatellite allele length at arginine vasopressin 1a receptor and oxytocin receptor loci. Proc. R. Soc. B Biol. Sci..

[5] Hannan AJ (2018). Tandem repeats mediating genetic plasticity in health and disease. Nat. Rev. Genet..

[6] Khademi E, Alehabib E, Shandiz EE, Ahmadifard A, Andarva M, Jamshidi J, Rahimi-Aliabadi S, Pouriran R, Nejad FR, Mansoori N, Shahmohammadibeni N, Taghavi S, Shokraeian P, Akhavan-Niaki H, Paisán-Ruiz C, Darvish H, Ohadi M (2017). Support for "Disease-Only" genotypes and excess of homozygosity at the CYTH4 primate-specific GTTT-repeat in Schizophrenia. Genet. Test Mol. Biomark..

[7] Bushehri A, Barez MR, Mansouri SK, Biglarian A, Ohadi M (2016). Genome-wide identification of human- and primate-specific core promoter short tandem repeats. Gene.

[8] Mohammadparast S, Bayat H, Biglarian A, Ohadi M (2014). Exceptional expansion and conservation of a CT-repeat complex in the core promoter of PAXBP1 in primates. Am. J. Primatol..

[9] Bilgin Sonay T, Carvalho T, Robinson MD, Greminger MP, Krützen M, Comas D, Highnam G, Mittelman D, Sharp A, Marques-Bonet T, Wagner A (2015). Tandem repeat variation in human and great ape populations and its impact on gene expression divergence. Genome Res..

[10] Afshar, H., Khamse, S., Alizadeh, F., Delbari, A., Najafipour, R., Bozorgmehr, A. *et al*. Evolving evidence on a link between the ZMYM3 exceptionally long GA-STR and human cognition. 2045–2322 Contract No: 1 (2020). 10.1038/s41598-020-76461-z PMC7655811 33173136

[11] Rosas I, Martínez C, Clarimón J, Lleó A, Illán-Gala I, Dols-Icardo O, Borroni B, Almeida MR, van der Zee J, Van Broeckhoven C, Bruni AC, Anfossi M, Bernardi L, Maletta R, Serpente M, Galimberti D, Scarpini E, Rossi G, Caroppo P, Benussi L, Ghidoni R, Binetti G, Nacmias B, Sorbi S, Piaceri I, Bagnoli S, Antonell A, Sánchez-Valle R, De la Casa-Fages B, Grandas F, Diez-Fairen M, Pastor P, Ferrari R, Álvarez V, Menéndez-González M (2020). Role for ATXN1, ATXN2, and HTT intermediate repeats in frontotemporal dementia and Alzheimer's disease. Neurobiol. Aging..

[12] Darvish H, Heidari A, Hosseinkhani S, Movafagh A, Khaligh A, Jamshidi J, Noorollahi-Moghaddam H, Heidari-Rostami HR, Karkheiran S, Shahidi GA, Togha M, Paknejad SM, Ashrafian H, Abdi S, Firouzabadi SG, Jamaldini SH, Ohadi M (2013). Biased homozygous haplotypes across the human caveolin 1 upstream purine complex in Parkinson's disease. J. Mol. Neurosci..

[13] Namdar-Aligoodarzi P, Mohammadparast S, Zaker-Kandjani B, Kakroodi ST, Vesiehsari MJ, Ohadi M (2015). Exceptionally long 5′ UTR short tandem repeats specifically linked to primates. Gene.

[14] Li QS, Sun Y, Wang T (2020). Epigenome-wide association study of Alzheimer’s disease replicates 22 differentially methylated positions and 30 differentially methylated regions. Clin. Epigenetics.

[15] Annear DJ, Vandeweyer G, Elinck E, Sanchis-Juan A, French CE, Raymond L (2021). Abundancy of polymorphic CGG repeats in the human genome suggest a broad involvement in neurological disease. Sci. Rep..

[16] Jiao B, Zhou L, Zhou Y, Weng L, Liao X, Tian Y (2020). Identification of expanded repeats in NOTCH2NLC in neurodegenerative dementias. Neurobiol. Aging..

[17] LaCroix AJ, Stabley D, Sahraoui R, Adam MP, Mehaffey M, Kernan K (2019). GGC repeat expansion and exon 1 methylation of XYLT1 is a common pathogenic variant in Baratela-Scott syndrome. Am. J. Hum. Genet..

[18] Ma D, Tan YJ, Ng AS, Ong HL, Sim W, Lim WK (2020). Association of NOTCH2NLC repeat expansions with Parkinson disease. JAMA Neurol..

[19] Sone J, Mitsuhashi S, Fujita A, Mizuguchi T, Hamanaka K, Mori K (2019). Long-read sequencing identifies GGC repeat expansions in NOTCH2NLC associated with neuronal intranuclear inclusion disease. Nat. Genet..

[20] Ajjugal Y, Kolimi N, Rathinavelan T (2021). Secondary structural choice of DNA and RNA associated with CGG/CCG trinucleotide repeat expansion rationalizes the RNA misprocessing in FXTAS. Sci. Rep..

[21] Kumutpongpanich T, Ogasawara M, Ozaki A, Ishiura H, Tsuji S, Minami N (2021). Clinicopathologic features of oculopharyngodistal myopathy with LRP12 CGG repeat expansions compared with other oculopharyngodistal myopathy subtypes. JAMA Neurol..

[22] Foroughan M, Wahlund LO, Jafari Z, Rahgozar M, Farahani IG, Rashedi V (2017). Validity and reliability of a bbreviated Mental TEst Score (AMTS) among older Iranian. Psychogeriatrics.

[23] Hodkinson H (1972). Evaluation of a mental test score for assessment of mental impairment in the elderly. Age Ageing.

[24] Folstein M (1975). A practical method for grading the cognitive state of patients for the children. J. Psychiatr. Res..

[25] Carpenter CR, Banerjee J, Keyes D, Eagles D, Schnitker L, Barbic D (2019). Accuracy of dementia screening instruments in emergency medicine: A diagnostic meta-analysis. Acad. Emerg. Med. Off. J. Soc. Acad. Emerg. Med.

[26] Sullivan KM, Dean A, Soe MM (2009). OpenEpi: A web-based epidemiologic and statistical calculator for public health. Public Health Rep..

[27] Drenos F, Kirkwood TB (2010). Selection on alleles affecting human longevity and late-life disease: the example of apolipoprotein E. PLoS One..

[28] Raj T, Shulman JM, Keenan BT, Chibnik LB, Evans DA, Bennett DA (2012). Alzheimer disease susceptibility loci: Evidence for a protein network under natural selection. Am. J. Hum. Genet..

[29] Thierry-Mieg D, Thierry-Mieg J (2006). AceView: A comprehensive cDNA-supported gene and transcripts annotation. Genome Biol..

[30] Persico AM, Levitt P, Pimenta AF (2006). Polymorphic GGC repeat differentially regulates human reelin gene expression levels. J. Neural Transm. (Vienna)..

[31] Sutcliffe JS, Nelson DL, Zhang F, Pieretti M, Caskey CT, Saxe D, Warren ST (1992). DNA methylation represses FMR-1 transcription in fragile X syndrome. Hum. Mol. Genet..

[32] Boivin M, Deng J, Pfister V, Grandgirard E, Oulad-Abdelghani M, Morlet B, Ruffenach F, Negroni L, Koebel P, Jacob H, Riet F, Dijkstra AA, McFadden K, Clayton WA, Hong D, Miyahara H, Iwasaki Y, Sone J, Wang Z, Charlet-Berguerand N (2021). Translation of GGC repeat expansions into a toxic polyglycine protein in NIID defines a novel class of human genetic disorders: The polyG diseases. Neuron.

[33] de Frutos-Lucas J, Frost N, Erickson KI, Serrano JM, Maestu F, Laws SM (2020). Does APOE genotype moderate the relationship between physical activity, brain health and dementia risk? A systematic review. Ageing Res. Rev..

[34] Karantzoulis S, Galvin JE (2011). Distinguishing Alzheimer’s disease from other major forms of dementia. Expert Rev. Neurother..

[35] Lin Y-F, Smith AV, Aspelund T, Betensky RA, Smoller JW, Gudnason V (2019). Genetic overlap between vascular pathologies and Alzheimer's dementia and potential causal mechanisms. Alzheimers Dement..

[36] Noori A, Mezlini AM, Hyman BT, Serrano-Pozo A, Das S (2020). Systematic review and meta-analysis of human Transcriptomics reveals Neuroinflammation, deficient energy metabolism, and Proteostasis failure across Neurodegeneration. Neurobiol. Dis..

