# Supplementary material for: Natural selection at the RASGEF1C (GGC) repeat in human and divergent genotypes in late-onset neurocognitive disorder
Source: Sci Rep. 2021 Sep 28;11:19235. doi: 10.1038/s41598-021-98725-y (PMC8479062; doi:10.1038/s41598-021-98725-y)
Supplement: Supplementary file 3 — Supplementary Legends. [file 41598_2021_98725_MOESM3_ESM.docx]

**Supplementary Files**

**Suppl. 1.**  Raw data of genotypes and alleles in the NCD cases

**Suppl. 2.** Raw data of genotypes and alleles in the Controls.
